# Feature selection for RNA cleavage efficiency at specific sites using the LASSO regression model in *Arabidopsis thaliana*

**DOI:** 10.1186/s12859-021-04291-5

**Published:** 2021-07-22

**Authors:** Daishin Ueno, Harunori Kawabe, Shotaro Yamasaki, Taku Demura, Ko Kato

**Affiliations:** Graduate School of Science and Technology, Nara Institute of Science and Technology, Ikoma, 630-0192 Japan

**Keywords:** RNA degradation, Degradome sequencing, LASSO

## Abstract

**Background:**

RNA degradation is important for the regulation of gene expression. Despite the identification of proteins and sequences related to deadenylation-dependent RNA degradation in plants, endonucleolytic cleavage-dependent RNA degradation has not been studied in detail. Here, we developed truncated RNA end sequencing in *Arabidopsis thaliana* to identify cleavage sites and evaluate the efficiency of cleavage at each site. Although several features are related to RNA cleavage efficiency, the effect of each feature on cleavage efficiency has not been evaluated by considering multiple putative determinants in *A. thaliana*.

**Results:**

Cleavage site information was acquired from a previous study, and cleavage efficiency at the site level (CS_site_ value), which indicates the number of reads at each cleavage site normalized to RNA abundance, was calculated. To identify features related to cleavage efficiency at the site level, multiple putative determinants (features) were used to perform feature selection using the Least Absolute Shrinkage and Selection Operator (LASSO) regression model. The results indicated that whole RNA features were important for the CS_site_ value, in addition to features around cleavage sites. Whole RNA features related to the translation process and nucleotide frequency around cleavage sites were major determinants of cleavage efficiency. The results were verified in a model constructed using only sequence features, which showed that the prediction accuracy was similar to that determined using all features including the translation process, suggesting that cleavage efficiency can be predicted using only sequence information. The LASSO regression model was validated in exogenous genes, which showed that the model constructed using only sequence information can predict cleavage efficiency in both endogenous and exogenous genes.

**Conclusions:**

Feature selection using the LASSO regression model in *A. thaliana* identified 155 features. Correlation coefficients revealed that whole RNA features are important for determining cleavage efficiency in addition to features around the cleavage sites. The LASSO regression model can predict cleavage efficiency in endogenous and exogenous genes using only sequence information. The model revealed the significance of the effect of multiple determinants on cleavage efficiency, suggesting that sequence features are important for RNA degradation mechanisms in *A. thaliana*.

## Background

RNA degradation is an important process for regulating gene expression in living organisms [1]. RNA degradation is mediated by deadenylation-dependent or endonucleolytic cleavage-dependent RNA degradation mechanisms [2]. In both degradation mechanisms, the final step in RNA digestion is catalyzed by exonucleases. Deadenylation-dependent RNA degradation has been studied in plants [3], and some proteins and related sequences were identified and analyzed; however, endonucleolytic cleavage-dependent RNA degradation has not been analyzed in detail.

Degradome sequencing methods, which are techniques for detecting truncated RNA ends using next-generation sequencing (NGS), have been developed to study the mechanisms underlying endonucleolytic cleavage–dependent RNA degradation [4–6]. Although these methods contribute to the identification of cleavage sites, the detected cleavage sites are biased toward the 3′ end of the transcript because of poly A selection in library preparation [6, 7]. In previous work from our group, we attempted to solve this problem by developing truncated RNA end sequencing (TREseq) in *A. thaliana* [8]. TREseq analysis showed high G nucleotide frequency around the cleavage sites; the cleavage sites were highly accumulated around the start and stop codons, and three-nucleotide periodicity was observed in the coding sequences (CDSs) [8, 9]. These tendencies are similar to ribosome movements, which are reported in ribosome profiling methods, suggesting that the translation process affects RNA cleavage [8–10]. In our previous study, we evaluated the relationships between single determinants and cleavage efficiency in *A. thaliana* using Pearson’s correlation analysis [11]. We found that cleavage efficiencies were related to several determinants (e.g., G nucleotide frequency around cleavage sites and RNA length). However, because Pearson’s correlation analysis can evaluate only one-to-one relationships, the effects of multiple putative determinants on cleavage efficiency remain to be elucidated.

To evaluate several features, multiple regression analysis has been performed using large feature sets [12, 13]. Sequence information (e.g., nucleotide sequence, codons, or amino acid usage) was used, and RNA abundance or RNA stability was predicted by a multiple regression model. However, multiple regression models can result in predictions with large variance, thereby affecting the accuracy of prediction in high-dimensional data [14]. To overcome the shortcomings of multiple regression in high-dimensional data, the Least Absolute Shrinkage and Selection Operator (LASSO) regression model was designed [15]. LASSO penalizes the absolute size of regression coefficients. Therefore, by setting as many coefficients as possible to zero, the objective variable is regressed with as few features as possible. Necessary features can thus be extracted according to the coefficient in the LASSO regression model. In a previous study, a LASSO regression model was applied to predicting the ribosome occupancy on RNA (i.e., the translation process) [16]. Sequence information was used, and approximately 60 features were reduced to a subset of 10–20 features. The results revealed that specific nucleotides or codon sequence patterns are important for the translation process. Although these integrated analyses using the LASSO regression model were used to predict the transcription (RNA expression level) or translation process (ribosome occupancy) [16, 17], little information is available in RNA degradation mechanism, especially for endonucleolytic cleavage–dependent RNA degradation in *A. thaliana*. Although some cleavage sites induced by microRNA were analyzed and could be predicted by computing RNA complementary sequences (nucleotide information) [18], the majority of cleavage sites did not appear to be induced by microRNA [8, 19, 20]. Therefore, the determinants of genome-wide RNA cleavage sites remain to be elucidated in *A. thaliana*.

To address this issue, we obtained cleavage sites throughout the genome and performed feature selection for cleavage efficiency using LASSO in *A. thaliana*. In the LASSO regression model, 155 features were selected, and the coefficients indicated that in addition to features around cleavage sites, features of the whole RNA were also important. We also confirmed the selected features in the LASSO regression model using a different regression model (Ridge regression), which addressed some of the problems of multiple regression models but did not decrease the number of features relative to LASSO. In addition, we attempted to predict cleavage efficiency in endogenous and exogenous genes using only sequence information, and the prediction accuracy was similar to that of the model using all features. These results suggest that sequence features in whole RNA and around cleavage sites are critical for determining the cleavage efficiency at each site in both endogenous and exogenous genes in *A. thaliana*.

## Materials and methods

### Plant material

*Arabidopsis thaliana* T87 cell suspension was obtained from Riken Cell Bank (Tsukuba,

Japan) and cultured in modified Murashige–Skoog medium, as described previously [21].

### Data processing for TREseq

Reads from cultured cells and seeds of *A. thaliana* ecotype Columbia-0 transformed with p35S::firefly luciferase (F-luc)::heat shock protein 18.2 terminator (HSPT) [11, 22] were acquired from previous TREseq analyses [8, 11] and mapped to the TAIR version 10 reference genome (www.arabidopsis.org) or the p35S::F-Luc::HSPT sequence using HISAT2. After mapping, the first nucleotide (5′ end) of each read was counted using BED files as described previously [9]. Cap RNA with more than 50 reads at each gene was used for RNA abundance information [9]. To estimate the cleavage efficiency at each site, the reads at each 5′ degradation intermediate normalized to RNA abundance were defined as the cleavage score at the site level (CS_site_). At the gene level, we defined the total CS_site_ value at each gene as the CS_gene_ value.

### Library construction for ribosome profiling

Ribosome-protected fragments (RPFs) were selected as described previously [23, 24]. In brief, *A. thaliana* T87 cells were harvested 3 days after inoculation and frozen in liquid nitrogen, followed by homogenization in extraction buffer (200 mM Tris–HCl, pH 8.5, 50 mM KCl, 25 mM MgCl_2_, 2 mM EGTA, 100 µg/ml heparin, 100 µg/ml cycloheximide, 2% polyoxyethylene 10-tridecyl ether, and 1% sodium deoxycholate), and centrifuged at 15,000 g for 10 min at 4 °C [23]. Cells were incubated with 6 μl RNase I (Thermo Fisher Scientific, MA, USA) for 30 min, and the reaction was stopped by addition of 10 μl RNase inhibitor (Thermo Fisher Scientific). A 26.25–71.25% sucrose density gradient buffer (200 mM Tris–HCl, pH 8.5, 200 mM KCl, and 200 mM MgCl_2_) was used to collect monosomes by sucrose density gradient centrifugation at 55,000 rpm for 50 min at 4 °C in an SW55 rotor (Beckman Coulter, CA, USA). After isolation of monosomes, RPFs were purified using the TruSeq Ribo Profile kit (Illumina). The libraries were sequenced on an Illumina NextSeq 500 (Illumina).

### Data processing for ribosome profiling

The adapter sequences were trimmed, and reads were mapped to the TAIR version 10 reference genome (www.arabidopsis.org) using the modified MOIRAI system [8, 9]. After mapping, the first nucleotide (5′ end) of each read was counted using BED files. To estimate the RPFs at each site, the average of RPF reads at the 5' end of each site normalized to RNA abundance was defined as ribosome occupancy at the site level (RO_site_). At the gene level, the total RO_site_ values at each gene were defined as RO_gene_ values.

### Analysis of cleavage sites using LASSO and Ridge regression

In TREseq analysis, cleavage sites are detected in approximately 2 million sites. To select reliable sites, we selected sites in the genes with > 20% cleaved sites relative to RNA length and whose CS_gene_ values were between the 5th and 95th percentile. In addition, we removed expected microRNA-induced cleavage sites using psRNATarget [18]. Data were separated into training and test sets (9: 1), and training data were used to construct the model.

### Explanatory variables in the model

**Fig. 1:**
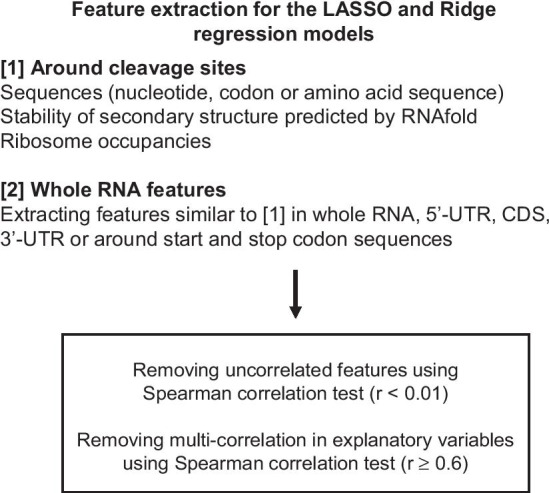
Feature extraction for models. Features related to sequences, stability of the secondary structure, and ribosome occupancy were extracted from around cleavage sites. For whole RNA features, similar features around cleavage sites were extracted from whole RNA, 5′-UTR, CDS, 3′-UTR, or around start and stop codon sequences. Uncorrelated features and multi-correlation in explanatory variables were removed.

In the model, features around the cleavage sites or features in whole RNA were extracted (Fig. 1). RNA sequence information (nucleotide, codon, and corresponding amino acid sequence) were obtained from the TAIR10 database (www.arabidopsis.org), and the minimum free energy (stability of secondary structure) was predicted using the RNAfold software (http://rna.tbi.univie.ac.at/) based on RNA nucleotide sequences [25]. In addition, in the ribosome profiling method used in this analysis, RO_site_ and RO_gene_ values were used to obtain ribosome occupancy information (Fig. 1). For the features around cleavage sites, we determined nucleotide, codon, and the corresponding amino acid sequences ± 30 nucleotides around the cleavage sites. A comprehensive search of the features around the cleavage sites was performed and nucleotide, codon, or corresponding amino acid frequencies were calculated. The window size was changed by one nucleotide (minimum length, 1 nucleotide; maximum length, 60 nucleotides) and the sliding window was shifted by one nucleotide. Because ribosome occupancy affects long-distance cleavage efficiency [26], the region was extended by ± 200 nucleotides around the cleavage site. In terms of secondary structure, because the minimum free energy of short RNA sequences cannot be predicted by RNAfold, the minimum window size was changed to 5 nucleotides (minimum length, 5 nucleotides; maximum length, 60 nucleotides), and the sliding window was shifted by 5 nucleotides.

**Fig. 2:**
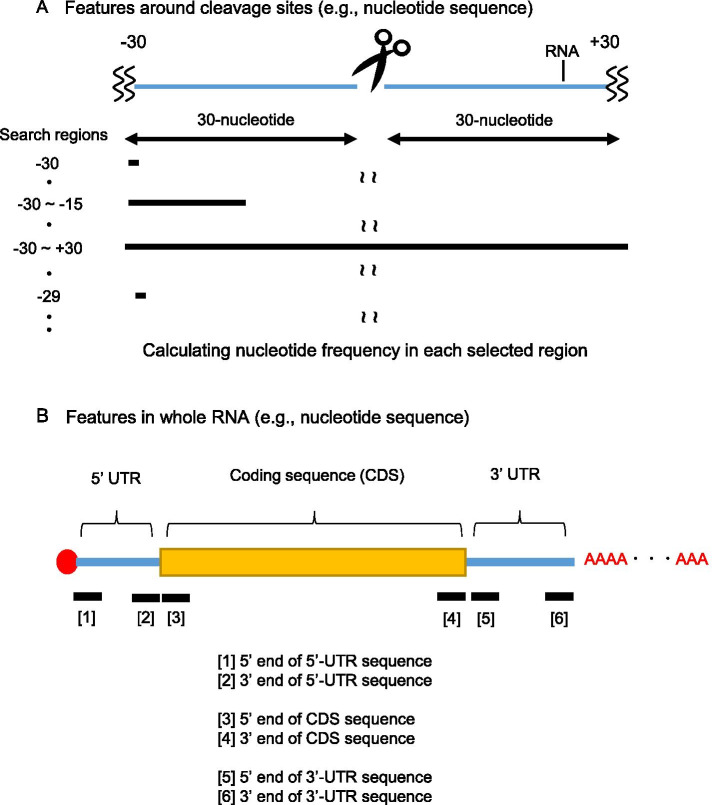
Example of an explanatory variable. Nucleotide sequences were used as explanatory variables (features) around cleavage sites (**a**) or in whole RNA (**b**). Search regions were comprehensively changed, and nucleotide frequencies were calculated in the selected region. Similar data processing was conducted in the whole RNA. Nucleotide frequencies were calculated in the 5′-UTR, CDS, 3′-UTR, or whole RNA and were also calculated in 50-nucleotide sequences from the 5’ or 3’ end of each region (5′-UTR, CDS, 3′-UTR, or whole RNA).

In terms of whole-RNA features, we extracted 5′-UTR, CDS, 3′-UTR, and whole RNA sequences from the TAIR10 database (www.arabidopsis.org) and calculated the nucleotide, codon, and corresponding amino acid frequencies. The minimum free energy was predicted using RNAfold in each region (5′-UTR, CDS, 3′-UTR, or whole RNA). The sum of RO_site_ values in each region (5′-UTR, CDS, 3′-UTR, or whole RNA) was used to obtain ribosome occupancy information. In addition, 50-nucleotide sequences were extracted from the 5’ or 3’ end of each region (5′-UTR, CDS, 3′-UTR, or whole RNA), and the nucleotide frequency information was added to the model. Because several codons around start codon appeared to be related to the translation process [27, 28], 10 codons or their corresponding amino acid sequences were extracted from 5’ or 3’ end of CDS, and the codons or corresponding amino acid frequencies were used for the model. An example of an explanatory variable using “nucleotide sequence” is shown in Fig. 2.

To remove multi-collinearity from explanatory variables, we calculated the Spearman rank correlation coefficient among features. If the correlation coefficient between features was ≥ 0.6, the feature with the highest correlation to the CS_site_ value was used for the model. In addition, explanatory variables that showed no correlation (r < 0.1) according to the Spearman coefficient were removed. Finally, 1,191 features were used for the LASSO and Ridge regression models. RNA sequence information can be obtained from the TAIR10 database, and the data processed in this analysis (cleavage sites and ribosome profiling information) are available in GitHub (https://github.com/daishin-ueno/LASSO_and_Ridge_regression/tree/main/Datasets).

### Construction of the LASSO and Ridge regression models

For feature selection, we used linear_model.Lasso or linear_model.Ridge from the Python package scikit-learn [29]. In the *i*-th observation, ${y}_{i}$ was the objective variable, and the cleavage efficiencies (CS_site_ values) and the vector ${\mathrm{x}}_{i}=\left({x}_{i1},{x}_{i2},\cdots ,{x}_{ip}\right)$ were the corresponding feature value set. The linear regression model of the cleavage efficiencies was defined as follows:

$${\widehat{y}}_{i}=\alpha +{\varvec{\upbeta}}\bullet {\mathbf{x}}_{i}=\alpha +\sum_{j=1}^{p}{\beta }_{j}{x}_{ij} ,$$

where ${\varvec{\upbeta}}\bullet {\mathbf{x}}_{i}$ represents the inner product of the vectors ${\varvec{\upbeta}}=\left({\beta }_{1},{\beta }_{2},\cdots ,{\beta }_{p}\right)$ and ${\mathbf{x}}_{i}$, ${\beta }_{j}$ is the coefficient for *j*-th feature, ${\alpha}$ is the intercept, and $p$ is the number of features.

The LASSO regression estimator uses the L1 regularization penalty:

$${{\varvec{\upbeta}}}_{LASSO}=\underset{{\varvec{\upbeta}}}{\mathrm{arg min}}\left\{{{\sum }_{i=1}^{N}\left({y}_{i}-{\widehat{y}}_{i}\right)}^{2}+\lambda {\sum }_{j=1}^{p}\left|{\beta }_{j}\right|\right\},$$

where $\lambda {\sum }_{j=1}^{p}\left|{\beta }_{j}\right|$ is the L1 regularization penalty on the coefficient ${\beta }_{j}$ and $\lambda \ge 0$ is the tuning parameter.

The Ridge regression estimator uses the L2 regularization penalty:

$${{\varvec{\upbeta}}}_{Ridge}=\underset{{\varvec{\upbeta}}}{\mathrm{arg min}}\left\{{{\sum }_{i=1}^{N}\left({y}_{i}-{\widehat{y}}_{i}\right)}^{2}+\lambda {\sum }_{j=1}^{p}{\beta }_{j}^{2}\right\},$$

where $\lambda {\sum }_{j=1}^{p}{\beta }_{j}^{2}$ is the L2 regularization penalty on ${\beta }_{j}$ and $\lambda \ge 0$ is the tuning parameter.

**Fig. 3:**
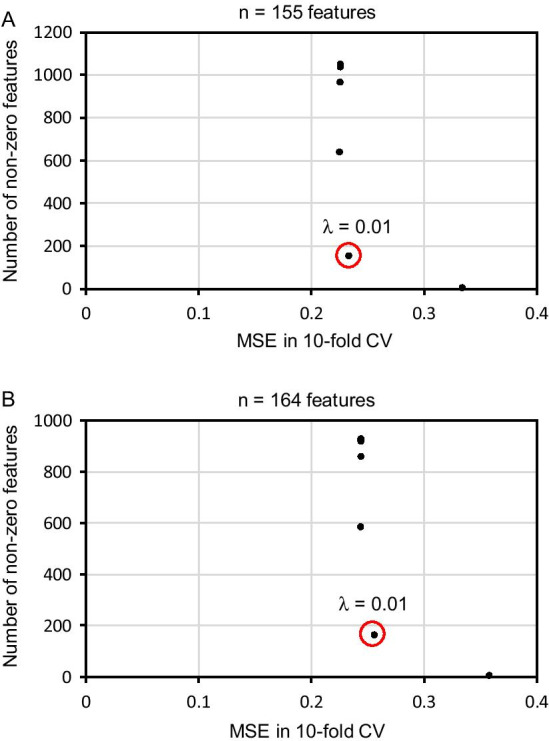
Optimizing the tuning parameters in the LASSO regression model. Average mean square error (MSE) was calculated in tenfold cross validation (tenfold CV) by changing the parameter λ in LASSO regression. The number of non-zero features in the LASSO regression model was also calculated, and the parameter λ was determined according to the average MSE and the number of non-zero features in the LASSO regression model (**a**). The same data processing approach was used for the LASSO regression model, which used only sequence information (**b**). The Y-axis indicates the number of non-zero features in the LASSO regression model. The X-axis indicates the average MSE in tenfold CV. λ (0.01) was used in each regression model

The tuning parameter λ was determined using training data. In LASSO regression, mean squared error (MSE) between predicted and measured CS_site_ values was calculated by changing the parameter λ (10^–10^–10^–1^) in tenfold cross validation [30] using model_selection.cross_val_score from the Python package scikit-learn [29, 31]. To increase interpretability in the LASSO regression model, we determined the parameter λ with a reduced number of features while maintaining MSE (Fig. 3a). The same data processing approach was used in the LASSO regression model, which was constructed using only sequence information (Fig. 3b). In Ridge regression, MSE between predicted and measured CS_site_ values was calculated by changing the parameter λ (10^–10^–10^–1^) in tenfold cross validation [30] using model_selection.cross_val_score from the Python package scikit-learn [29, 31]; we determined that λ = 10^5^ where MSE was smallest in the range 10^–10^–10^–1^. Source codes for the LASSO or Ridge regression model using linear_model.Lasso or linear_model.Ridge from the Python package scikit-learn are available at GitHub (https://github.com/daishin-ueno/LASSO_and_Ridge_regression/tree/main/Source_code).

## Results and discussion

### Data processing in the LASSO regression model

**Fig. 4:**
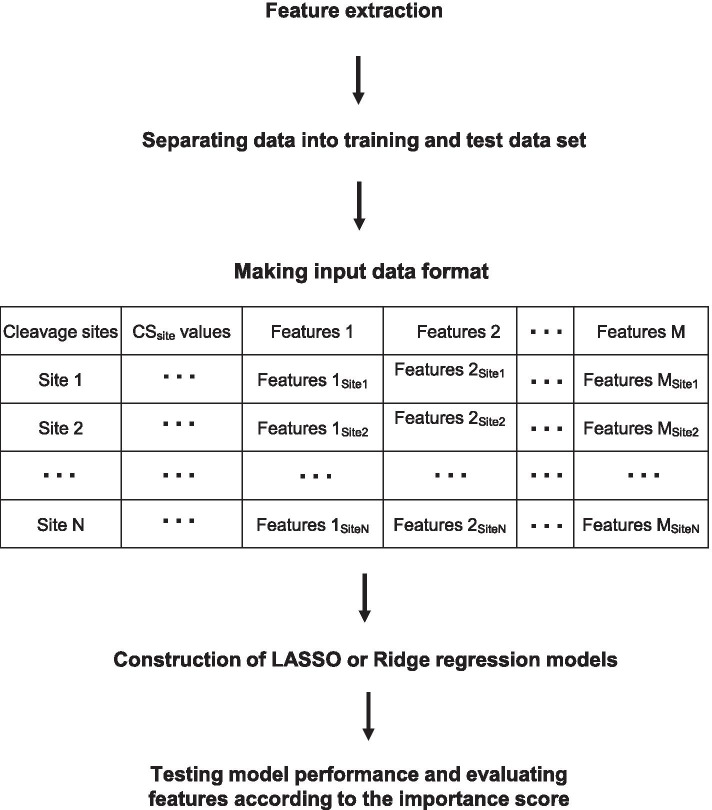
Data processing in the LASSO and Ridge regression models. Sequences (nucleotide, codon, or corresponding amino acid sequence), stability of secondary structures, and ribosome occupancy information was obtained, and features with multi-collinearity among explanatory variables or no correlation to the objective variable were removed from the feature extraction process. Cleavage sites (CS_site_ values) were divided into training and test data sets, and input data were formatted. Subsequently, the LASSO or Ridge regression model was constructed using the training dataset. Finally, model performance was evaluated using test data, and features of non-zero coefficients were estimated according to the importance score (coefficients in the LASSO or Ridge regression model)

For the objective variable, indicators of cleavage efficiency at the site level (CS_site_ value) were calculated using our previous TREseq data [8], with the number of reads at each cleavage site normalized to RNA abundance. Explanatory variables (features) can be divided into three categories: sequence (nucleotide, codon, or corresponding amino acid frequency), stability of secondary structure, and ribosome occupancy (Fig. 1). For the explanatory variables (features), RNA sequences were obtained from the TAIR10 database, and nucleotide, codon, and corresponding amino acid frequencies were acquired. Minimum free energies of secondary structures were calculated using the RNAfold software based on RNA nucleotide sequences. In addition, ribosome occupancy information (DRA010802) was obtained under the conditions used for our previous TREseq data [8], and the RO_site_ value (the number of ribosome-protected fragments at the site level, normalized against RNA abundance) was used for model. Because multi-collinearity among features negatively affects the prediction accuracy, we removed features with high correlations between explanatory variables. In addition, features that showed no correlation between objective and explanatory variables according to Spearman’s correlation test (r < 0.01) were removed from the feature extraction process (Fig. 4). To obtain reliable cleaved sites, we selected genes with > 20% cleaved sites relative to RNA length. The total numbers of analyzed genes and sites were 1,107 and 429,185 sites, respectively. Sites were separated into training and test data sets (Table 1), and the CS_site_ value was used as the objective variable. Input data were formatted using CS_site_ values and features (Fig. 4) and are available in GitHub (https://github.com/daishin-ueno/LASSO_and_Ridge_regression/tree/main/Datasets/Final_input_data_for_model_construction). The LASSO or Ridge regression model was then constructed using training data, and its performance was evaluated using test data. Features of non-zero coefficients were selected, and each feature was estimated according to its importance score in the model (Fig. 4).

**Table 1:** Training and test data in the LASSO and Ridge regression models

	Training	Test
Number of cleavage sites	395,375	43,742
Number of genes	996	111

### Prediction of CS_site_ value using the LASSO regression model

**Fig. 5:**
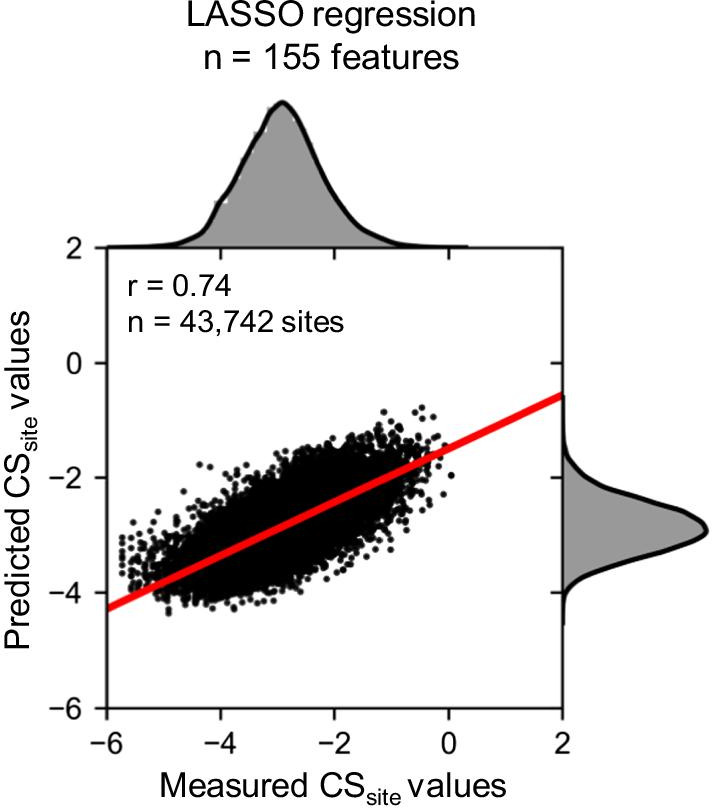
Prediction of the CS_site_ value using the LASSO regression model. The model was constructed using training data, and Pearson’s correlation coefficient was calculated using test data. The X-axis indicates the measured CS_site_ value in TREseq and the Y-axis indicates the predicted CS_site_ value in the LASSO regression model. Histograms above and to the right of each plot show the distribution of measured and predicted CS_site_ values, respectively

**Fig. 6:**
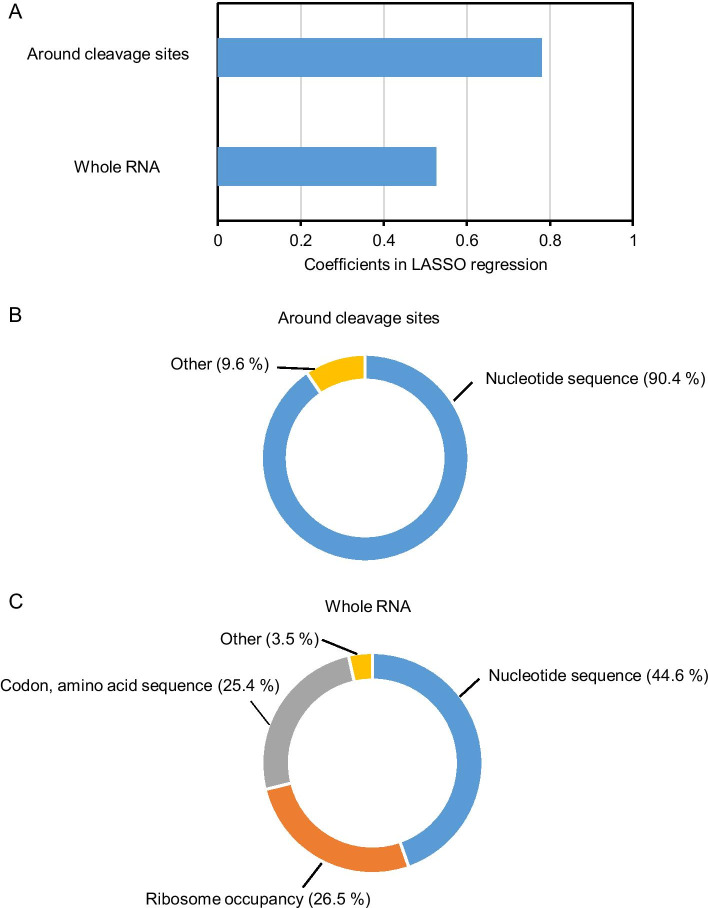
Positive coefficients in the LASSO regression model. Positive coefficient features around cleavage sites and in whole RNA were extracted (**a**). The proportions of coefficients were categorized into features around the cleavage sites (**b**) and features of the whole RNA (**c**).

The prediction accuracy (Pearson’s correlation coefficient) was calculated using test data; the correlation coefficient was r = 0.74 (Fig. 5). Features with a coefficient of zero were removed, which decreased the number of features from 1,191 to 155. The resultant 155 features predicted the cleavage efficiency. These features were divided into positive (contributing to increased cleavage efficiency) and negative (contributing to decreased cleavage efficiency) groups according to the correlation coefficient. In the positive coefficient (effect) group, the correlation coefficient of whole RNA features around cleavage sites was 0.78 (59.7%) and that of whole RNA features was 0.53 (40.3%) (Fig. 6a). Nucleotide sequences accounted for approximately 90% of the positive coefficients in the features around cleavage sites (Fig. 6b). This result is consistent with those of previous studies reporting that nucleotide frequency around cleavage sites has a positive effect on CS_site_ values [8, 9, 11], and suggests that sequence features are major determinants of CS_site_ values in the positive coefficient features. When we focused on the positive coefficients of whole RNA features, ribosome occupancy, codon, and corresponding amino acid sequence accounted for approximately 50% (Fig. 6c). These results suggest that the translation process (codon, corresponding amino acid sequence, or ribosome occupancy) has a positive effect on CS_site_ value in features of the whole RNA, but not in features around cleavage sites.

**Fig. 7:**
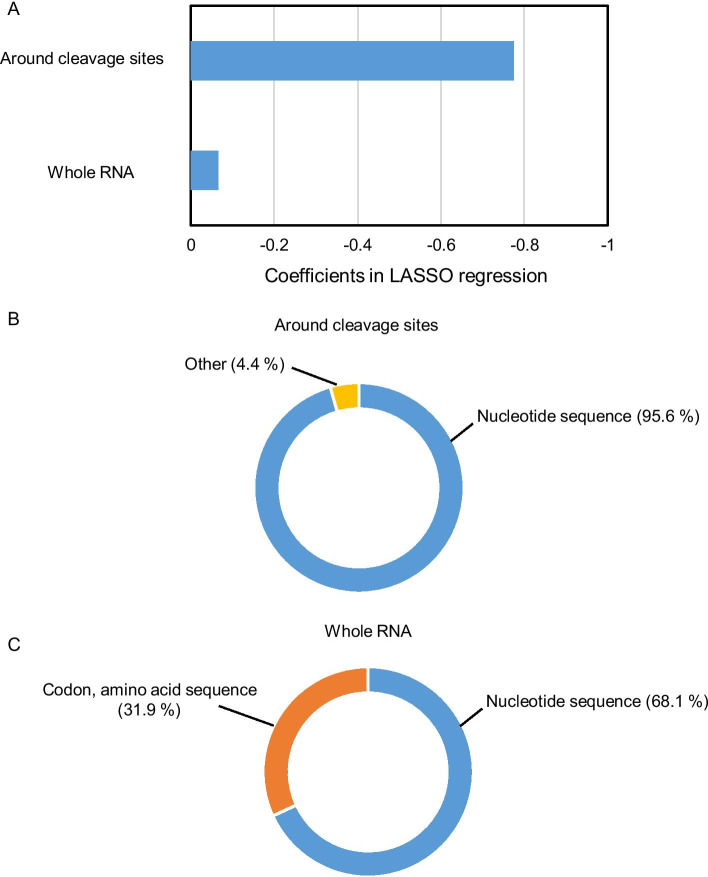
Negative coefficients in LASSO regression. Negative coefficient features around cleavage sites and features in whole RNA were extracted (**a**). Detailed features were categorized, and the proportions of coefficient in the LASSO regression model in features around cleavage sites (**b**) and features in whole RNA (**c**) were calculated

In the negative coefficient group, whole RNA features were also related to cleavage efficiency; however, the majority of features were around cleavage sites (Fig. 7a). Analysis of the features around the cleavage sites indicated that nucleotide frequency was a major determinant of CS_site_ values in the negative coefficient group (Fig. 7b), which is similar to the observation in the positive coefficient features around cleavage sites (Fig. 6b). Among whole RNA features, codon or corresponding amino acid sequences were selected in addition to nucleotide sequences in the negative coefficient group (Fig. 7c). Considering that some nucleotide sequence patterns have an effect on ribosome occupancies [16], the nucleotide sequences in whole RNA features seemed to be involved in the translation process and to affect cleavage efficiency at the site level (Figs. 6c, 7c). These tendencies were also observed after selecting the five most positive or negative features based on the correlation coefficient in the LASSO regression model (Tables 2, 3). In particular, a G nucleotide frequency of − 4 to + 2 (positive) and a G nucleotide frequency of + 4 to + 5 (negative) were selected. These results are consistent with the nucleotide frequency around the cleavage sites because G nucleotide frequency was high from the − 3 to + 1 position, whereas it was low around the + 4 position, as reported in a previous TREseq analysis [8]. Taken together, these results indicate that the 155 features explained the CS_site_ value in the LASSO regression model, and whole RNA features (e.g., translation process) were related to cleavage efficiency, in addition to nucleotide frequency around cleaved sites.

**Table 2:** Positive coefficient features in the LASSO regression model

Features (positive)	Coefficient
Ribosome occupancy in RNA	0.135
GG frequency around cleavage sites − 4 to + 2	0.108
G frequency around cleavage sites at + 1	0.090
G frequency around cleavage sites − 2 to + 1	0.067
G frequency around cleavage sites − 1 to + 3	0.045

**Table 3:** Negative coefficient features in the LASSO regression model

Features (negative)	Coefficient
G frequency around cleavage sites + 4 to + 5	− 0.061
G frequency around cleavage sites + 8 to + 14	− 0.052
U frequency around cleavage sites at − 2	− 0.051
C frequency around cleavage sites at + 4	− 0.050
A frequency around cleavage sites + 17 to + 19	− 0.042

### Confirmation of selected features using a different model

**Fig. 8:**
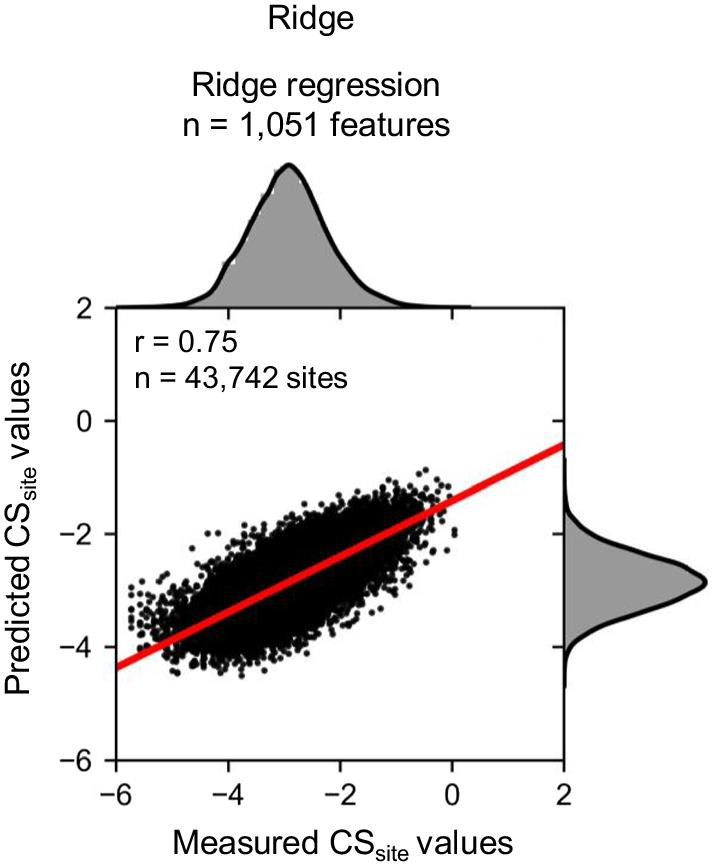
Prediction of the CS_site_ value using the Ridge regression model. The model was constructed using training data, and Pearson’s correlation coefficient was calculated using test data. The X-axis indicates the measured CS_site_ values in TREseq and the Y-axis indicates the predicted CS_site_ values in the Ridge regression model. Histograms above and to the right of each plot show the distribution of measured and predicted CS_site_ values, respectively

**Fig. 9:**
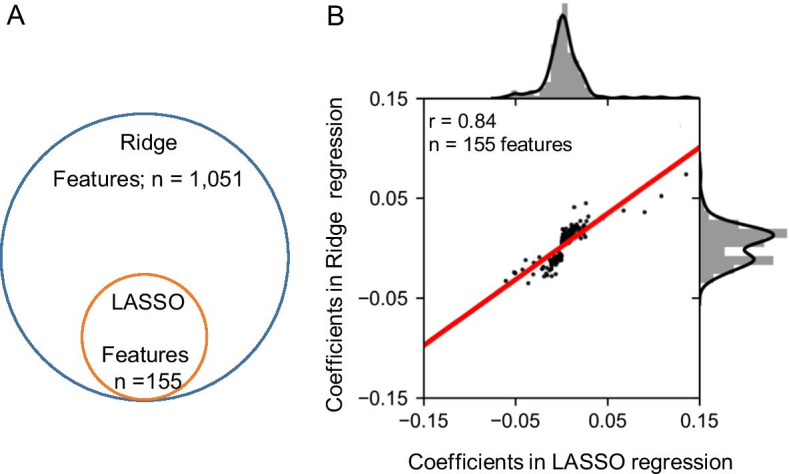
Confirmation of selected features in the LASSO regression model using the Ridge regression model. There were 155 common features in the LASSO and Ridge regression models (**a**). Pearson’s correlation coefficient was calculated using the common features in both models (**b**)

The advantages of the proposed LASSO regression model include reduction of the number of features (explanatory variables) and increased interpretability. On the other hand, the LASSO regression model cannot select several features if they have similar correlations to the objective variable. Hence, it is possible that some important features for cleavage efficiencies were removed from the LASSO regression model. To overcome this limitation, we needed to confirm that similar trends would be observed if we used a different model. Therefore, to confirm the importance of the features selected in the LASSO regression model, we performed an experiment using the Ridge regression model. We constructed the Ridge regression model based on data processing in the LASSO regression model. The prediction accuracy of Ridge was first tested using Pearson’s correlation coefficient, which showed that the prediction accuracy was comparable to that of the LASSO regression model (Fig. 8). The features with a coefficient of zero were removed, and the 1191 features were reduced to 1,051 features (Fig. 9a). We also calculated the correlation coefficient of feature importance, which is common in the LASSO and Ridge regression models, using Pearson’s correlation coefficient, and similar tendencies were observed in both models (Fig. 9b). In addition, when the five most positive or negative features were selected (Tables 4, 5), nucleotide frequency around the cleavage sites and whole RNA features related to the translation process were selected. These results suggest that features selected in the LASSO regression model are reliable.

**Table 4:** Positive coefficient features in the Ridge regression model

Features (positive)	Coefficient
Ribosome occupancy in RNA	0.074
GG frequency around cleavage sites − 4 to + 2	0.052
GG frequency around cleavage sites − 1 to + 1	0.045
GG frequency around cleavage sites − 3 to − 1	0.041
G frequency around cleavage sites − 2 to + 1	0.037

**Table 5:** Negative coefficient features in the Ridge regression model

Features (negative)	Coefficient
AA frequency around cleavage sites − 1 to + 2	− 0.035
G frequency around cleavage sites + 4 to + 5	− 0.033
GU frequency around cleavage sites + 4 to + 6	− 0.028
A frequency around cleavage sites − 1 to + 1	− 0.028
UG frequency around cleavage sites + 1 to + 2	− 0.027

### Predicting CS_site_ values using only sequence information

**Fig. 10:**
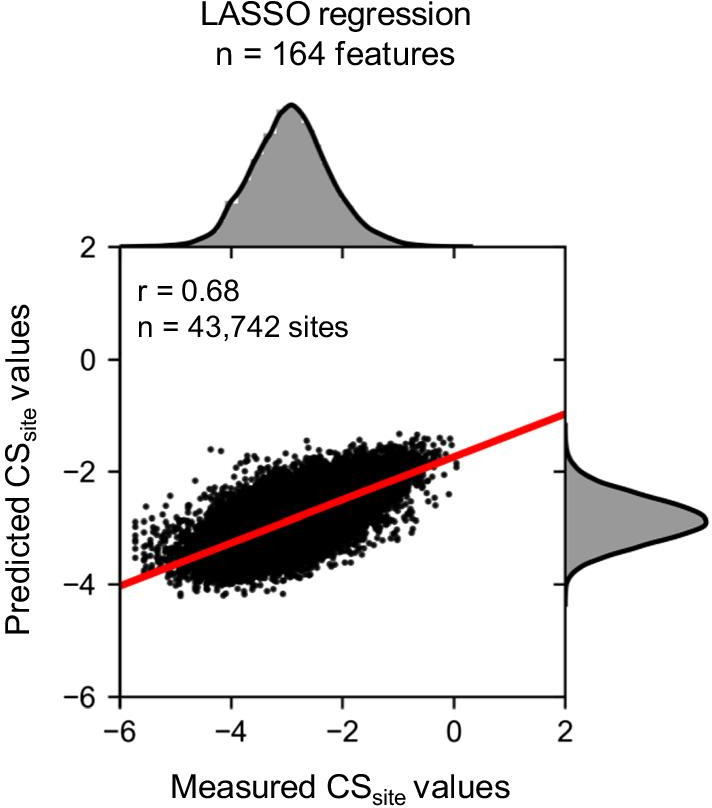
Prediction of the CS_site_ value using the LASSO regression model (only sequence information). The model was constructed using training data, and Pearson’s correlation coefficient was calculated using test data. The X-axis indicates the measured CS_site_ values in TREseq and the Y-axis indicates the predicted CS_site_ values in the LASSO regression model. Histograms above and to the right of each plot show the distribution of measured and predicted CS_site_ values, respectively

Although ribosome occupancy had the highest positive coefficient in the LASSO regression model, most coefficients were related to sequence information. In addition, ribosome occupancy was explained by nucleotide or codon sequence in a previous study [15]. Thus, we hypothesized that we could predict cleavage efficiency at each site using only sequence information. We removed features related to ribosome occupancy or secondary structure information and re-constructed the LASSO regression model. Prediction accuracy (Pearson’s correlation coefficient) was calculated using test data; the correlation coefficient was r = 0.68 (Fig. 10). These results indicate that cleavage efficiency at the site level could be explained using only sequence information.

### Validation of the LASSO regression model through prediction of exogenous genes

**Fig. 11:**
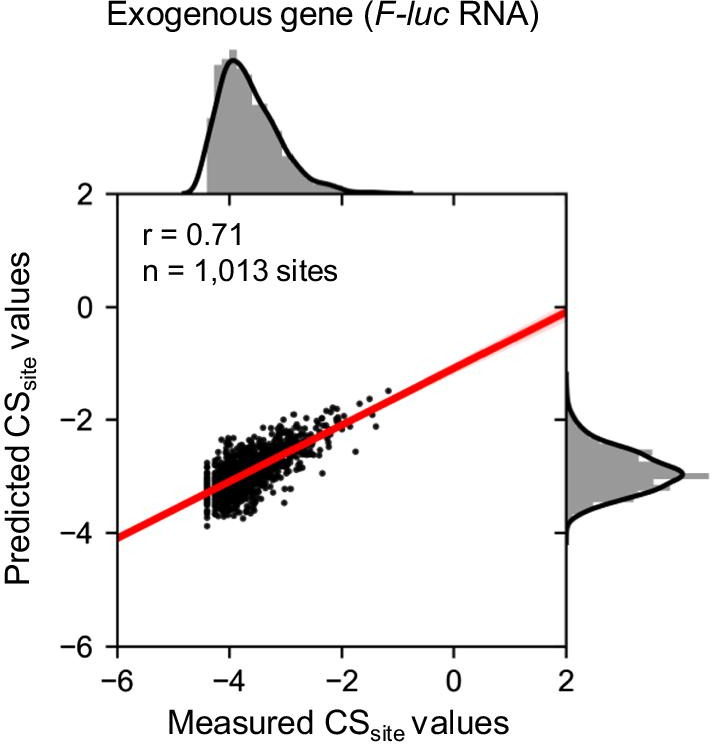
Validation of the LASSO regression model through prediction of CS_site_ values in exogenous genes. The LASSO regression model constructed using only sequence information was used to predict CS_site_ values in *F-luc* RNA (exogenous gene). The X-axis indicates the measured CS_site_ values in TREseq and the Y-axis indicates the predicted CS_site_ values in the LASSO regression model

The LASSO regression model was constructed using endogenous genes. If this model could explain cleavage efficiency in plant cells, we hypothesized that cleavage efficiency could be predicted in exogenous genes in *A. thaliana.* We obtained the CS_site_ values of the *Firefly luciferase* (*F-luc*) gene, which was inserted into the *A. thaliana* genome (DRA009373) [11, 22]. Because ribosome profiling information for the *F-luc* gene was lacking, we used a model constructed using only sequence information (Fig. 10). We predicted CS_site_ values in *F-luc* RNA and calculated the Pearson's correlation coefficient between measured and predicted CS_site_ values. The prediction accuracy was r = 0.71 (Fig. 11). These results suggest that the selected features for CS_site_ values in the LASSO regression model are reliable, and RNA cleavage efficiency at the site level in both endogenous and exogenous genes can be predicted using only sequence information in *A. thaliana*.

## Conclusions

We conducted feature selection using the LASSO regression model in *A. thaliana*. The model revealed the significance of the effect of multiple determinants on cleavage efficiency at each site, and suggests that whole RNA features are important for determining cleavage efficiency in addition to features around the cleavage sites. The selected features in the LASSO regression model were validated using a different method, and this model could predict cleavage efficiency in both endogenous and exogenous genes using only sequence information. These results indicate that mathematical models can predict cleavage efficiency at the site level in transgenes in plants, providing new insight into the importance of sequence features for RNA degradation mechanisms in *A. thaliana.*

## Data Availability

TREseq reads of cultured cells and *F-luc* RNA are available in the DDBJ Sequence Read Archive (DRA) database under accession numbers DRA005995 (https://ddbj.nig.ac.jp/DRASearch/study?acc=DRP003990) and DRA009373 (https://ddbj.nig.ac.jp/DRASearch/submission?acc=DRA009373). Ribosome profiling reads in *A. thaliana* are available under the accession number DRA010802 (https://ddbj.nig.ac.jp/DRASearch/submission?acc=DRA010802). Processed data in this study are available at GitHub (https://github.com/daishin-ueno/LASSO_and_Ridge_regression).
